# The efficacy of formal auditory training in children with (central) auditory processing disorder: behavioral and electrophysiological evaluation

**DOI:** 10.1016/S1808-8694(15)30525-5

**Published:** 2015-10-18

**Authors:** Renata Alonso, Eliane Schochat

**Affiliations:** 1 MSc in Rehabilitation Sciences - FMUSP, Graduate Student in Rehabilitation Sciences - FMUSP; 2 Associate Professor - Speech and Hearing Therapy - FMUSP. Center for Research and Teaching of Speech and Hearing Therapy, Physical therapy and Occupational Therapy - Medical School of the University of São Paulo

**Keywords:** hearing, acoustic stimulation, neuronal plasticity, p300 evoked potential

## Abstract

Long Latency Auditory Evoked Potentials can be used to monitor changes in the Central Auditory Nervous System after Auditory Training.

**Aim:**

The aim of this study was to investigate the efficacy of Auditory Training in children with (Central) Auditory Processing Disorder, comparing behavioral and electrophysiological findings before and after training.

**Materials and Methods:**

twenty nine individuals between eight and 16 years of age with (Central) Auditory Processing Disorder - diagnosed by behavioral tests - were involved in this research. After evaluation with the P300, the subjects were submitted to an Auditory Training program in acoustic booth and, at the end, a new evaluation of (central) auditory processing and a new recording of P300.

**Results:**

The comparison between the evaluations made before and after the Auditory Training showed that there was a statistically significant difference among P300 latency values and also among behavioral test mean values in evaluation of (central) auditory processing.

**Conclusion:**

P300 appears to be a useful tool to monitor Central Auditory Nervous System changes after Auditory Training, and this program was effective in the rehabilitation of the auditory skills in children with (Central) Auditory Processing Disorder.

## INTRODUCTION

Auditory processing disorders (APD) comprise a complex and heterogeneous set of alterations frequently associated with a number of auditory and sensorial deficits^1^, ^2^.

Auditory training (AT) is a broadly used means to intervene upon individuals with APD. Various studies have indicated that AT may have a positive impact on the temporal processing of children experiencing linguistic and learning difficulties^3^, ^4^, ^5^. AT is indicated to improve auditory system function in the resolution of acoustic signals^1^.

Musiek et al.^6^ have defined AT as a set of conditions and/or tasks designed to activate the auditory system and other systems associated with it, aimed at producing beneficial changes to auditory behavior and the central auditory nervous system (CANS).

Auditory training optimizes neural circuits by increasing the number of neurons involved in the process, changing neural temporal synchronicity, and increasing the number of synaptic connections^7^.

Changes to the CANS after AT are based on the plasticity of the central nervous system^8^, ^9^. The latter can be defined as changes to neural cells to better meet the immediate environmental requirements; these changes are usually associated with behavior modification^10^,^11^. These changes depend, among other variables, on the quality and consistence of the stimuli provided1 and involve changes to neural connections and activity in multiple levels of the central auditory pathway^12^.

Several authors have reported in the usefulness of auditory evoked potentials (AEP) in monitoring changes to the CANS secondary to AT^6^,^8^,^11^.

Jirsa^13^ has stated that AEPs offer significant value in assessing progress in individuals submitted to treatment programs. According to this author, neurophysiologic changes occur prior to behavior changes introduced as a result of therapeutic intervention. Evidence indicates that AEPs outperform traditional behavior assessment when it comes to evaluating the progress of individuals submitted to treatment programs.

Kraus et al.^14^ have reported that changes to CANS neurophysiology secondary to AT can be measured and monitored through long latency evoked auditory potentials (LLEAP).

P300 is an endogenous LLEAP made up by a positive wave with post-stimulation latency of approximately 300ms indicative of activity in brain areas responsible for specific functions such as attention and memory^15^, ^16^, ^16^, ^17^.

P300 is frequently evoked in tasks of auditory discrimination, in which the subject has to respond to target stimuli presented randomly and in small number between other more frequent stimuli - oddball paradigm^18^,^21^.

Tremblay et al.^22^ studied the P300 on normal individuals and concluded that, after auditory discrimination therapy, there was a reduction on the latency of wave P300 among tested subjects.

A few previously mentioned studies identified the existence of changes in the CANS after stimulation or AT. It is utterly important that these studies are confirmed and further information is gathered so that the effectiveness of certain AT tasks is proven through long latency electrophysiological measurements (e.g.: P300).

This study aims to verify the effectiveness of AT in children with APD through behavior assessment and P300 LLEAP measurement.

## MATERIALS AND METHOD

This study was approved by our institution's ethics committee under permit 707/06.

Twenty-nine subjects with APD were enrolled in the study. Sixteen were males and 13 females. Ages ranged from 8 to 16 years.

Enrollment criteria: tone threshold audiometry, logoaudiometry, and impedance test results within normal values; absence of present and past auditory complaints; brainstem auditory evoked potential (BAEP) within normal range and altered results in at least two central auditory processing behavior assessment tests.

Parents and guardians to the children enrolled in the study signed a free informed consent term prior to the beginning of the tests.

Subjects with confirmed normal impedance test, tone audiometry, and logoaudiometry results were submitted to behavior tests to diagnose APD.

Behavior tests consisted of two monotic and two dichotic tests. In the monotic test set, we used the Brazilian Portuguese version of synthetic sentence identification with ipsilateral competing message (SSI-ICM), through which figure and ground skills for verbal sounds and selective attention were evaluated, and speech test with white noise used to assess selective attention and auditory closure. In dichotic testing, we used non-verbal directed attention tests to verify selective attention during a task of binaural separation and the staggered spondaic word (SSW) test, in which subjects were presented with 40 sequences of 4 two-syllable paroxytone words, among which two words are presented in a competitive condition.

Electrophysiological tests were then carried out in a silent room. BAEP (to verify brainstem integrity) and P300 data were recorded.

The following were the parameters used for P300 acquisition: monaural acoustic stimulation (tone burst with a 20 ms plateau and 5 ms rise/fall) for frequencies of 1000 and 1500 Hz at 75 dBNA; analysis time of 800ms; filter for 1 to 30 Hz; sensitivity of 100 μV. Five-hundred stimuli were delivered, 75% of which were frequent (1000 Hz) and 25% rare (1500 Hz). Rare and frequent stimuli were presented randomly (oddball paradigm).

Electrodes were positioned on the vertex (Cz) and on each of the ear sides (A1 for left ear and A2 for right ear); the ground electrode was placed on the contralateral ear in relation to the ear being tested. Right and left ears were assessed separately.

Before the electrodes were placed in the above mentioned sites, abrasive paste was applied on these areas so as to reduce electric impedance between skin and electrodes to below 5 ohms.

P300 was identified as a wave of positive polarity with post-stimulation latency of approximately 300 ms; it is obtained by subtracting the tracing corresponding to rare stimuli in the corresponding tracing of frequent stimuli. Amplitude and latency values for the P300 were obtained. P300 analysis was performed by the author and another researcher (blind examiner) so as to eliminate bias in the assessment of the data obtained before and after AT.

After the APD diagnosis was confirmed by behavior tests and P300 analysis, subjects were submitted to an AT program based on the procedure proposed by Chermak and Musiek^23^ and Musiek and Chermak^24^ and validated by Musiek and Schochat^4^.

AT was delivered in eight 50-minute weekly sessions. Patients and their guardians were advised to perform the tasks at home. Each subject was handed a list of the tasks they had to perform at home.

The difficulty level of each task comprised in the AT program was manually set for each test and session, in order to maintain a success/failure rate of approximately 70/30%^4^. The tasks on each AT session were planned one week in advance and, in such plan, the results obtained and tasks performed by the subjects in the previous sessions were considered, so as to minimize the possibility of one same task being repeated in future sessions.

At the end of the AT program, another central auditory processing behavior evaluation was performed. One month after this behavior review, P300 waves were acquired again. This one-month waiting period was established to ensure the stability of the neurophysiologic changes resulting from AT.

Impedance tests were conducted using a Grason-Stadler GSI-33 middle ear analyzer, while tone audiometry, logoaudiometry, central auditory processing behavior, and AT were performed with an audiometer of the same brand, model GSI-61, and a Siemens soundproof booth. A Bio-Logic Traveler Express device was used for BAEP and P300 assessment.

Data analysis was performed with non-parametric tests Wilcoxon and Mann-Whitney and parametric tests ANOVA and paired Student's T-test. Level of statistical significance was set at 5%. The confidence interval technique was also use to complete the descriptive analysis.

## RESULTS

No statistically significant differences were found between right and left ears in behavior and electrophysiological assessment, showing that tested ear is not a difference-generating factor. Thus, the values for both ears were considered altogether.

On the first electrophysiological examination (done prior to AT), nine of the 29 enrolled patients had no detectable P300 wave. Four of these nine did not have detectable P300 waves on their right ears, another four when stimuli were applied to the left ear, and one in either of the ears.

On the second electrophysiological examination (done after AT), only one of the 29 subjects did not have detectable P300 waves on his/her right ear.

In the cases where no match was found for P300, the amplitude value considered for statistical analysis was 0 (zero) μV and the latency value ascribed was 500ms (simulation).

The 500ms value was defined as a function of the maximum latency value found among subjects in our study (462ms) and the maximum values reported by other studies done with patients of the same age range: 530ms for Polish et al.^25^; 540ms for Oades et al.26 and 450ms for Hirayasu et al.^17^.

Table 1 shows that statistically significant differences were found for P300 latency values before and after AT. Mean latency values also decreased significantly in the last P300 assessment.

**Table 1:** Descriptive latency measurements on initial and final electrophysiological evaluation

Latency (ms)	Initial	Final
Mean	382,66	351,21
Median	366	342
Standard Deviation	65,76	47,13
CV	17,2%	13,4%
Quartile 1	334	324,5
Quartile 3	420	375,5
Size	58	58
CI	16,92	12,13
p-value	0,001*	

Legend: CV - coefficient of variation;

CI - confidence interval;

* p-value - deemed statistically significant

Table 2 reveals that no statistically significant differences were found between mean amplitude values before and after AT. Even though no statistically significant differences were found, the mean amplitude values were lower in the second evaluation than in the first.

**Table 2:** Descriptive amplitude measurements on initial and final electrophysiological evaluation

Amplitude (μV)	Initial	Final
Mean	5,50	6,74
Median	4,67	5,65
Standard Deviation	4,55	4,59
CV	82,8%	68,2%
Quartile 1	2,63	3,54
Quartile 3	7,61	9,46
Size	58	58
CI	1,17	1,18
p-value	0,178	

Legend: CV - coefficient of variation;

CI - confidence interval;

p-value - deemed statistically significant

Table 3 shows that statistically significant differences were found in all central auditory processing behavior tests when comparing before and after AT results.

**Table 3:** Behavioral assessment results - pre and post auditory training

	SSI-ICM			Speech with noise			DNV			SSW		
	Initial		Final	Initial		Final	Initial		Final	Initial		Final
Mean	66,2%		86,4%	68,8%		80,1%	8,62		10,93	72,0%		89,2%
Median	70,0%		90,0%	68,0%		80,0%	9,00		12,00	72,5%		92,5%
Standard Deviation	17,8%		13,1%	10,8%		7,0%	2,52		1,78	12,0%		11,0%
CV	26,8%		15,1%	15,6%		8,8%	29,2%		16,2%	16,6%		12,4%
Quartile 1	50,0%		80,0%	64,0%		76,0%	7,00		11,00	65,0%		87,5%
Quartile 3	80,0%		100%	76,0%		84,0%	11,00		12,00	82,4%		95,0%
Size	58		58	58		58	58		58	58		58
CI	4,6%		3,4%	2,8%		1,8%	0,65		0,46	3,1%		2,8%
p-value	<0,001*			<0,001*			<0,001*			<0,001*		

Legend: SSI-ICM - synthetic sentence identification with ipsilateral competing message;

DNV - directed attention non-verbal dichotic test;

SSW - staggered spondaic word test;

CV - coefficient of variation;

CI - confidence interval;

* p-value - deemed statistically significant

Among the children diagnosed with APD in the initial assessment, 72.4% had normal auditory processing test results after AT.

## DISCUSSION

Previous studies have used LLEAP to assess neurophysiologic changes occurred after AT and observed improvements in amplitude, latency and/or wave morphology after auditory stimulation^27^, ^28^, ^29^, ^30^, ^31^.

In this study, statistically lower mean latency values were found when before and after AT mean latency values were compared.

No statistically significant differences were found when comparing mean amplitude values before and after AT, but greater amplitude values were identified in electrophysiological evaluation after AT.

The data gathered on P300 amplitude and latency suggest that auditory stimulation performed during AT introduces changes to the CANS, as monitored in the P300 waves.

The results described herein are in agreement more specifically with the findings published by Jirsa^27^ on 20 children with APD and ages ranging between 9 and 12 years submitted to a 14-session AT program. After auditory stimulation, the children experienced P300 reduced latency and increased amplitude. However, differently from our study, the increases in amplitude found by Jirsa^27^ were statistically significant. Although no statistically significant differences were found in the mean values of amplitude in our study before and after AT, the after AT P300 values were considerably greater. Statistically significant difference could possibly be detected in our amplitude values if subjects had attended a greater number of AT sessions, as done by Jirsa^27^ (14 sessions).

Kozlowski et al.^32^ observed a reduction in P300 latency in a 9-year-old child diagnosed with APD after four months of speech therapy with auditory stimulation. In spite of the differences in time and means of delivering auditory stimulation between our study and Kozlowski et al.^32^, the authors did not report significant differences in P300 amplitude after auditory stimulation, as seen in our study.

Our results are also in agreement with the findings reported by Gil^33^, in which significant reductions in latency were found in a group of 14 individuals with hearing loss after they underwent eight AT sessions in a soundproof booth. Gil^33^ offered AT in patterns rather similar to those used in our study, and the author could not find any statistically significant differences in P300 amplitude when comparing pre and post AT results either.

The electrophysiological data found in our study suggest that, after AT, neurophysiologically beneficial changes probably occur in the CANS. These changes probably occur in response to sensorial experiences, and manifest themselves through improved neural synchronicity and/or nerve cell specificity differentiation and reorganization and/or increase in the number of neurons responding to sensorial information^34^; these changes are based on the plasticity of the central nervous system.

P300 latency had a more pronounced improvement than amplitude in the subjects submitted to auditory stimulation, as also seen in other studies. This shows that P300 latency, when compared to amplitude, is a more sensitive measurement of the potential for neurophysiologic change secondary to auditory stimulation programs.

CNS plasticity is the basis for success on AT, and LLEAP - P300 specifically - is a useful tool in monitoring CANS changes occurring after AT6, as confirmed by the electrophysiological data raised in this study.

Many other studies also verified the use of P300 in monitoring CANS changes after AT, showing that evoked potentials can be utilized to follow CANS changes resulting from auditory stimulation^11^,^15^,^36^, ^37^, ^37^, ^38^, ^39^.

All behavior assessment test results presented statistically significant differences before and after AT. These results show that the auditory training performed in this study improved auditory figure and ground skills, the association between auditory and visual stimuli, auditory closure, and binaural association/separation of speech and non-speech sounds.

Our study's findings are in agreement with the results reported by Zalcman and Schochat^39^ in a study conducted within the same scope as ours. Zalcman and Schochat^39^ also found statistically significant differences when they compared central auditory processing test results before and after AT, with improvements observed in all tests after auditory training. The authors reported that environmental aspects, more specifically the AT program, stimulated the neural structures connected to trained auditory skills, as also seen in the results reported in this study.

Schochat et al.^38^ analyzed children with APD submitted to AT within the same parameters of our study. In behavior assessment performed after AT, improvements were also found in all test results with statistically significant differences when comparing the pre and post AT mean number of right and wrong answers on SSI-ICM, speech with noise, non-verbal dichotic and SSW tests, as also seen in our study.

In our study we have also found that 72.4% of the subjects evolved to presenting normal test results when evaluated for auditory processing after AT.

Many other studies report improvements on behavior tests in populations with APD after subjects are submitted to AT programs^22^,^27^, ^28^, ^29^,^32^,^34^. Such improvements were also verified in our study, in which a significantly higher number of right answers in auditory processing tests was found after subjects had undergone AT.

The results found in this study, namely the improvement offered by AT upon various trained auditory skills, are directly related to the ability the central nervous system has to change itself when faced with stimulation, a capacity that may be referred to as neural plasticity. Therefore, we may state that the AT program used in this study led to beneficial changes upon the central nervous system, as confirmed by the improved performance subjects had on the tests used to assess behavior and by the changes observed on electrophysiological measurements after AT.

## CONCLUSION

The auditory training program used in this study was effective in rehabilitating the altered auditory skills of children with ADP. P300 proved to be useful in monitoring the changes occurred on the central auditory nervous system after auditory training.
